# Web‐based training intervention to increase physical activity level and improve health for adults with intellectual disability

**DOI:** 10.1111/jir.12984

**Published:** 2022-10-10

**Authors:** S. Fjellstrom, E. Hansen, J. Hölttä, M. Zingmark, A. Nordström, M. Lund Ohlsson

**Affiliations:** ^1^ Department of Health Sciences, The Swedish Winter Sport Research Centre Mid Sweden University Östersund Sweden; ^2^ The Faculty of Education and Arts Nord University Bodø Norway; ^3^ Health and Social Care Administration, Municipality of Östersund Östersund Sweden; ^4^ Department of Epidemiology and Global Health, Faculty of Medicine Umeå University Umeå Sweden; ^5^ Department of Health Sciences, Faculty of Medicine Lund University Lund Sweden; ^6^ Department of Public Health and Clinical Medicine, Section for Sustainable Health Umeå University Umeå Sweden; ^7^ School of Sports Science UiT The Arctic University of Norway Tromsø Norway

**Keywords:** e‐training, health equity, health promotion, online training, physical activity, quality of life

## Abstract

**Background:**

Individuals with intellectual disability (ID) are less physically active, have a higher body mass index (BMI) and are at greater risk for cardiovascular diseases (CVDs) than people without ID. The purpose of the study was to explore the effectiveness of a web‐based training programme, consisting of 150 min of activity per week, on the health of people with ID.

**Method:**

Participants with ID living in supported accommodation (*n* = 28, 48% female, age = 36.4 ± 9.56 years) participated in a web‐based training programme, consisting of a combination of exercises (endurance, strength balance and flexibility) of moderate intensity, 50 min, three times per week for 12 weeks. The body composition and waist circumference (WC) were measured, and questionnaires were used to assess enjoyment, quality of life (QoL) and physical activity (PA) level. Descriptive statistics and pairwise comparison pre and post intervention were carried out.

**Results:**

A total of 22 out of 28 participants completed the 12‐week training intervention with 83% mean attendance of training sessions. The intensity of the PA level increased and a decrease in fat mass of 1.9 ± 2.4 kg, *P* < 0.001 and WC of 3 ± 5 cm, *P* = 0.009 were observed. Enjoyment of training sessions was 3.9 out of 5, and no differences in QoL were found.

**Conclusion:**

A web‐based training programme is an effective tool for improving health parameters of people with ID and offers a new way for caregivers to enhance the PA for the target group.

## Introduction

About 1–3% of the population have an intellectual disability (ID) (Daily *et al*. 2000; Maulik *et al*. 2011), which is characterised by a limitation in intellectual functioning, that is, an intelligence quotient (IQ) of <70, and in adaptive functioning (World Health Organisation 2007; Shogren & Turnbull 2010). People with ID are less physically active than their counterparts without ID, and the health aspects of this target group are alarming. About 45% of people with ID are overweight, and 20% have obesity, which is 50% more compared to peers without ID (Doody & Doody 2012; Emerson *et al*. 2016); hence, there is a high risk of them developing a cardiovascular disease (CVD) or type 2 diabetes. An inactive lifestyle also affects quality of life (QoL), and in general people with ID rate their QoL much lower than people without ID (World Health Organisation 2000). However, with increased physical activity (PA), it is possible to promote QoL and prevent CVDs (Bondar *et al*. 2020), and according to the World Health Organisation (WHO), 75% of all CVDs can be prevented by following the recommended 150–300 min per week of PA on a moderate intensity (Elinder *et al*. 2010).

There are many barriers to PA for people with ID, such as transportation issues, a lack of awareness of options, financial limitations, negative support from caregivers or parents and a lack of clear policies for involvement in regular PA in residential and day‐care service programmes (Bodde & Seo 2009). Due to the COVID‐19 pandemic, the amount of PA has decreased dramatically although Sweden seems to be one of the least affected countries (Tison *et al*. 2020). In general, people living in rural area are less physically active than people living in urban areas (Kurti *et al*. 2015) and the barriers to exercising are even harder to overcome, for example, transport issues and a lack of options for exercising. To overcome some of the barriers, innovative solutions to increase the level of PA of people with ID have been investigated. One example is the use of digital tools, such as smartphone reminders regarding exercise and education in health, which have been shown to significantly improve the level of PA of people with ID, in both rural and urban areas (Kurti *et al*. 2015; Perez‐Cruzado & Cuesta‐Vargas 2017). Other digital interventions, such as web‐based training, have been successful in increasing the level of PA in other target groups, without disabilities. Web‐based training has also shown promising results in older adults, in whom positive changes in waist circumference (WC) and a decrease in fat mass have been observed, along with an increased level of PA (Ballin *et al*. 2020a; Ballin *et al*. 2020b).

People with ID can experience signs of aging from their thirties, and a comparison with older adults is therefore feasible (Connolly 2006). Increasing the level of PA is one way to delay signs of ageing. Previous studies have also shown that web‐based training is both cost‐efficient and increases the level of PA (Jahangiry *et al*. 2017; Linke *et al*. 2019). Moreover, this kind of training makes it easier to overcome some of the barriers observed in previous studies; since the training can be conducted at home, transport is no longer an issue, and using digital tools makes it easier for caregivers to provide support. Digital tools are more frequently used nowadays; however, as far as the authors know, exercise interventions with digital support for people with ID have not yet been investigated. Some studies have investigated virtual reality‐based exercise programmes to improve the physical fitness of people with ID, and positive outcomes in the resting heart rate have been observed (Lotan *et al*. 2010).

WHO updated their recommendations for PA in the autumn of 2020. Adults with disability are now specifically mentioned, and the recommendation for PA is for them to complete 150–300 min of moderate intensity or 75–150 min vigorous PA per week compared to the prior recommendations of only 150 min of moderate intensity per week (World Health Organisation 2010; World Health Organisation 2020). Previous studies have shown promising results from combining resistance and cardiovascular training for the target group. Exercise interventions for people with ID demonstrate different results in health outcomes (Calders *et al*. 2011; Bouzas *et al*. 2019). In a systematic review, is it shown that combined endurance and strength training show improvements in body composition (BC) measurements, for example, a decrease in fat mass, whereas other studies do not show significant changes in BC measurements but instead show changes in functional outcomes, such as the sit‐to‐stand test and grip strength (Bouzas *et al*. 2019). Measuring BC and WC is an easy way to perform a risk assessment for metabolic syndrome and is a good predictor of CVDs (Zeng *et al*. 2012; Matsushita *et al*. 2014). They are therefore essential to include when analysing the effects of training.

The global disabilities action plan highlights the importance of specific actions (World Health Organisation 2018). WHO raised serious concerns regarding health equity for people with disabilities as well as their health, and one of the objectives of the action plan is to remove barriers and improve accessibility to health services. Given that physical inactivity is the fourth leading risk factor for mortality and that people with ID are more sedentary than the general population, methods to prevent physical inactivity and promote PA need to be established. Interventions to promote PA have been tailored for people with ID (Bouzas *et al*. 2019; Hassan *et al*. 2019). However, to the best of the authors' knowledge, no previous studies have evaluated web‐based training programmes. Therefore, the objective of this study was to explore the feasibility and effectiveness of a web‐based training programme, 150 min of PA per week, for 12 weeks, for people with ID. A secondary outcome was to evaluate the enjoyment of the training programme and QoL.

## Method

### Study design

A total of 28 men and women with mild to moderate ID (American Psychiatric Association 2013) volunteered to participate in a 12‐week web‐based exercise intervention including three training sessions per week, each lasting 50 min. The intervention is described using a Consensus on Exercise Template (CERT) with 16 items (Slade *et al*. 2016) (see Table S1). The participants received a baseline assessment prior to the intervention. Follow‐up assessments were made after the intervention period. The intervention was performed in autumn 2020, during the COVID‐19 pandemic. The study was pre‐approved by the Swedish Ethical Review Authority (Dnr 2019‐06495 and Dnr 2020‐02607).

### Participants

To recruit participants, video‐based information was recorded and sent out to all supported living accommodations (a residence for people receiving support and services in accordance with the *Swedish Act Support and Service for Persons with Certain Functional Impairments*, LSS) in all municipalities in the county of Jämtland, Sweden, classified as rural areas (3.5 inhabitants/km^2^). This information consisted of an information session where the researchers presented the tests and training in pictures and videos. The inclusion criteria were: adults (18–60 years) with mild to moderate ID, according to DSM‐5 (American Psychiatric Association 2013), living in supported living accommodations, inactive according to the WHO's definition of less than 150 min of PA per week (Tremblay *et al*. 2017; World Health 2018), able to walk and able to understand video‐based exercise instructions. Caregivers at the supported living accommodation made a first judgement of whether the people in their accommodation would match the inclusion criteria. The level of PA was controlled by help from the caregivers, and no participant was estimated to reach 150 min of PA per week. Thereafter, researchers visited the supported living accommodation and participants were assisted by caregivers to understand the written information (adapted in cooperation with a special education teacher, written in simple language and using image support) and could ask the researchers questions. The participants decided for themselves whether they wanted to participate in the study regardless of whether other people in the same supported living accommodation agreed to participate. They agreed by signing a consent form.

### Intervention

Participants received a web‐based training programme, which was pre‐recorded, through a commercially available platform MyMOWO (https://www.mymowo.com, Virtual Gym Sweden AG, Sweden). Prior to the intervention, the training programme was tested by 10 persons with ID from a high school to determine the appropriate starting level and their advice were implemented. The training programme followed the WHO guidelines of 150 min per week of moderate intensity PA. The intensity was applied using the OMNI‐Walk/Run Scale of Perceived Exertion (Utter *et al*. 2004), a rating of perceived exertion with 1 as the lowest and 10 as the highest intensity. The participants could see pictures that described the different intensity levels and tried to reach 6–7 on the scale. The training frequency was three times a week including 50 min per session (2 × 25 min). The participants could choose if they wanted to perform the two sessions as one session or if they wanted to split the sessions 2 × 25 and perform one session in the morning and one in the evening for example. No instructions concerning rest in between were given. A combination of strength and endurance exercises was included in the training programme along with balance and flexibility. The programme was modified for people with ID in cooperation with a special education teacher, a physiotherapist and the training instructors working with the commercial platform. The adjustments made were (1) fewer instructions and talking from the instructor, (2) exercises that required easier coordination, (3) longer periods performing the same exercise before changing, (4) breaks with instructions to drink water and the instructor drinking water and (5) a timer showing the remaining time of the training session. A personal trainer demonstrated the web‐based training programme. The equipment needed for the exercises was a computer/reading tablet or mobile phone to be able to watch the videos. No other training equipment was needed. The participants were able to choose if they wanted to do the exercise in a group with others or by themselves and they performed the training in their home and/or in a community facility where they live. Different progression levels were applied to be able to meet the participants' requirements, for example, they could choose from jumping/walking to be able to meet the moderate intensity level. The exercise was unsupervised; however, the caregivers working with the participants were invited to participate if they wanted to. See Video S1 for a brief example of the training programme. Twice during the intervention period, researchers held digital motivational talks with participants in groups, after 4 and 8 weeks. The purpose of the talks was to identify any difficulties at an early stage and overcome the barriers together. Participants, assisted by caregivers, filled in a protocol after they had participated in each completed training session. This measured adherence and registered adverse events. Training attendance was defined as the number of sessions attended divided by the number of sessions planned. The caregivers working with the participants were also informed about the protocol and reminded the participants to complete it. Extra PA and/or exercise performed was not registered.

### Outcomes

The feasibility of the training programme was measured using the adherence rate, that is, the rate of completion of the training sessions as reported. Interviews with a sample of participants and caregivers were performed pre and post intervention and will be presented in a separate paper.

The effect of the training programme was assessed by measuring BC, WC, QoL and the PA level. The BC was measured using a Body Composition Analyser (InBody 270, Seoul, South Korea), which utilised Bioelectrical Impedance Analysis (BIA) (2016 InBody Co., Ltd). The BC was measured at the same time of the day prior to and post intervention, made by the same test leader, in the same indoor location (similar temperature 20–25°C). Electrodes were cleaned before measurement, and the measurement was repeated until the system accepted the result. Participants were instructed to step onto the scale with light clothes (only one layer such as t‐shirts and tights), bare feet and with their feet aligned with the foot electrodes. They were instructed to hold their hands on the handles with their thumbs placed on the oval electrodes and their arms stretched out from their body. Lean muscle mass and fat mass were measured in kilograms (kg). Participants received no instructions concerning intake of food and drinks. The WC was measured in centimetres, midway between the lowest rib and the iliac crest with a measuring tape (Ross *et al*. 2020). The BC and WC were assessed prior to and post the intervention.

QoL was evaluated using the questionnaire Manchester Short Assessment of Quality of Life (MANSA) (Priebe *et al*. 1999), a short version of the Lancashire Quality of Life Profile (Oliver 1991). The questionnaire is a reliable tool to measure QoL. Two studies present a Cronbach's alpha score of 0.83 and 0.81 for a population with mental illness (Björkman & Svensson 2005; Eklund & Sandqvist 2006). The MANSA consists of 12 questions in total. The questions are answered using a satisfaction scale of life as a whole, work, financial situation, friendships, leisure activities, accommodation, people that the participant lives with (or living alone), relationship with family, physical and mental health. Satisfaction is rated on a 7‐point rating Likert scale (1 = *negative*, 7 = *positive*). Two of the questions are to be answered *yes* or *no*. The MANSA was assessed both prior to and post the intervention.

The level of PA was measured using the short form of a questionnaire assessing PA levels over the last 7 days: the International Physical Activity Questionnaire (IPAQ‐SF). Studies have shown the reliability of the IPAQ‐SF for measuring the level of PA, with it obtaining similar results to objective tests, for example, accelerometery or pedometry (Craig *et al*. 2003; Kurtze *et al*. 2008; Cleland *et al*. 2018). It has also been shown to be successful in measuring the PA of people with ID (McKeon *et al*. 2013). The questionnaire consists of seven questions addressing PA in everyday life. Days, hours and minutes of vigorous PA, moderate activity, walking and sitting during a week were reported. The measures result in metabolic equivalent of task (MET), and, in accordance with the recommendations in IPAQ scoring protocol, three levels of PA (low, moderate and high) were reported, which is detailed elsewhere (Craig *et al*. 2003) (http://www.ipaq.ki.se/). To assist the participants with communication difficulties, images of PA topics were used. This is a reliable and valid tool for motivating people with ID to take part in a questionnaire process with a reliability score of 0.87 (Reid *et al*. 2009; Latorre Roman *et al*. 2014). Caregivers assisted the participants with reporting their levels of PA. The IPAQ‐SF was assessed both prior to and post the intervention.

The PA enjoyment was assessed using the questionnaire Physical Activity Enjoyment Scale (PACES), which is a reliable and valid tool (Kendzierski & Decarlo 1991). The elements of the PACES questionnaire are shown in Table 1. The PACES consists of 18 questions in total. Seven items are reverse‐coded. Enjoyment is rated using a 5‐point Likert scale (1 = *hate it*; 5 = *enjoy it*). Higher values indicate greater levels of enjoyment. The PACES was assessed post the intervention.

**Table 1:** Physical Activity Enjoyment Scale (PACES) 18 items

No.	Item
1	I enjoy it; I hate it
2	I feel bored; I feel interested
3	I dislike it; I like it
4	I find it pleasurable; I find it unpleasurable
5	I am very absorbed in this activity; I am not at all absorbed in this activity
6	It's no fun at all; It's a lot of fun
7	I find it energising; I find it tiring
8	It makes me depressed; It makes me happy
9	It's very pleasant; Its very unpleasant
10	I feel good physically while doing it; I feel bad physically while doing it
11	It's very invigorating; It's not at all invigorating
12	I am very frustrated by it; I am not at all frustrated by it
13	It's very gratifying; It's not at all gratifying
14	It's very exhilarating; It's not at all exhilarating
15	It's not at all stimulating; It's very stimulating
16	It gives me a strong sense of accomplishment; It does not give me any sense of accomplishment
17	It's very refreshing; It's not at all refreshing
18	I felt as though I would rather be doing something else; I felt as though there was nothing else I would rather be doing

### Statistical method

Descriptive statistics were calculated, and values are presented as means and standard deviations (SD). The Shapiro–Wilk test for normality was used to assess whether data were normally distributed. For normally distributed data, the paired‐samples *t*‐test was used to assess changes within the group, and the Wilcoxon signed‐rank test was used for non‐normal distributed data. A *P*‐value of <0.05 was considered statistically significant. Effect sizes were computed using Cohen's d for the paired‐samples *t*‐test, and the *R* effect size (*R*) was reported for the Wilcoxon signed‐rank test, classified as small (0.2 ≤ d < 0.5), medium (0.5 ≤ d < 0.8) and large (d ≥ 0.8) (Cohen 1988; Cohen 1992; McGrath & Meyer 2006). All statistical analyses were performed using IBM SPSS Statistics for Windows version 25 (IBM Corporation, Armonk, NY, USA).

## Results

### Participation in and adherence to the intervention

Of the 28 participants, four dropped out during the intervention period: one due to injury; the participant sustained a rib injury while biking, one due to a lack of Internet connection and two due to lack of motivation. After the training period, an additional two participants dropped out because they did not want to do the follow‐up tests; thus, 22 participants completed the study and performed web‐based training for 12 weeks. These 22 participants performed a mean of 30 of the 36 planned training sessions, that is, the mean adherence rate to the training sessions was 83%. Two additional participants did not perform a post‐test of the IPAQ questionnaire due to a lack of energy.

### Participant characteristics

The characteristics of the participants are presented in Table 2. Of the participants, 54.5% were males, and 45.5% were females. The mean age of the participants was 36.2 ± 10 years, and no age difference between the sexes was observed (*P* = 0.06).

**Table 2:** Descriptive characteristics of the participants pre‐training (age, height and weight) and comparison of body composition measures, PA level and QoL; pre‐intervention and post‐intervention

	Total (*n* = 22)					Females (*n* = 10)					Males (*n* = 12)				
Variables	Pre‐training	Post‐training	Change (pre–post)	*P*	Effect size	Pre‐training	Post‐training	Change (pre–post)	p	Effect size	Pre‐training	Post‐training	Change (pre‐‐post)	p	Effect size
Age (years)	36.2 ± 10	‐		‐	‐	40.5 ± 10.6	‐		‐	‐	32.6 ± 8.1	‐		‐	‐
Height (cm)	163.0 ± 14.0	‐		‐	‐	154.0 ± 11.1	‐		‐	‐	170.2 ± 12.0	‐		‐	‐
Weight (kg)	81.6 ± 22.4	‐		‐	‐	75.0 ± 14.2	‐		‐	‐	87.2 ± 26.9	‐		‐	‐
Total lean mass (kg)	27.6 ± 6.3	28.0 ± 6.4	0.4 ± 1.1	.12	0.35	23.3 ± 4.3	24 ± 5.2	0.8 ± 1.37	0.10	0.59	31.3 ± 5.3	31.3 ± 5.5	0.3 ± 0.7	0.9	0.04
Total fat mass (kg)	31.6 ± 17.4	29.7 ± 17.6	−1.9 ± 2.4	<0.001*	0.82	32.4 ± 12.2	31.0 ± 13.1	−1.4 ± 3.1	0.19	0.45	31 ± 21.3	28.6 ± 21	−2.4 ± 1.6	<0.001*	1.5
Waist circumference (cm)	101.0 ± 1.9	98 ± 1.8	−3.0 ± 4.8	0.009*	0.62	98.6 ± 1.6	95.3 ± 1.4	−3.3 ± 4.0	0.03*	0.83	102.9 ± 2.2	100.3 ± 2.2	−2.7 ± 5.5	0.12	0.48
BMI (kg/m2)	31.1 ± 8.3	30.5 ± 8.2	−0.6 ± 0.8	0.006*	0.70	32.3 ± 8.4	32.2 ± 8.3	−0.1 ± 0.9	0.70	0.10	30.1 ± 8.4	29.2 ± 8.2	−0.9 ± 0.5	<0.001*	1.7

Values are presented with mean ± standard deviation or number and percentage, *n* (%), and significant results are marked with asterisk (*).

### Effects of the intervention on BC, WC, PA level, QoL and enjoyment

In terms of the body composition measurements, the fat mass decreased significantly by a mean of 1.9 ± 2.4 kg, *P* < 0.001. This was most prominent for men and not significant for women. The lean body mass was similar before and after the intervention; for both men and women, no significant changes were observed. Waist circumference decreased by a mean of 3 ± 4.8 cm, *P* = 0.009. The waist circumference of the women decreased by a mean of 3.3 ± 4 cm and the men 2.7 ± 5.5 cm, indicating significant changes for women, *P* = 0.03, d = 0.8, but not for men, *P* = 0.1, d = 0.5 (Table 2).

**Figure 1:**
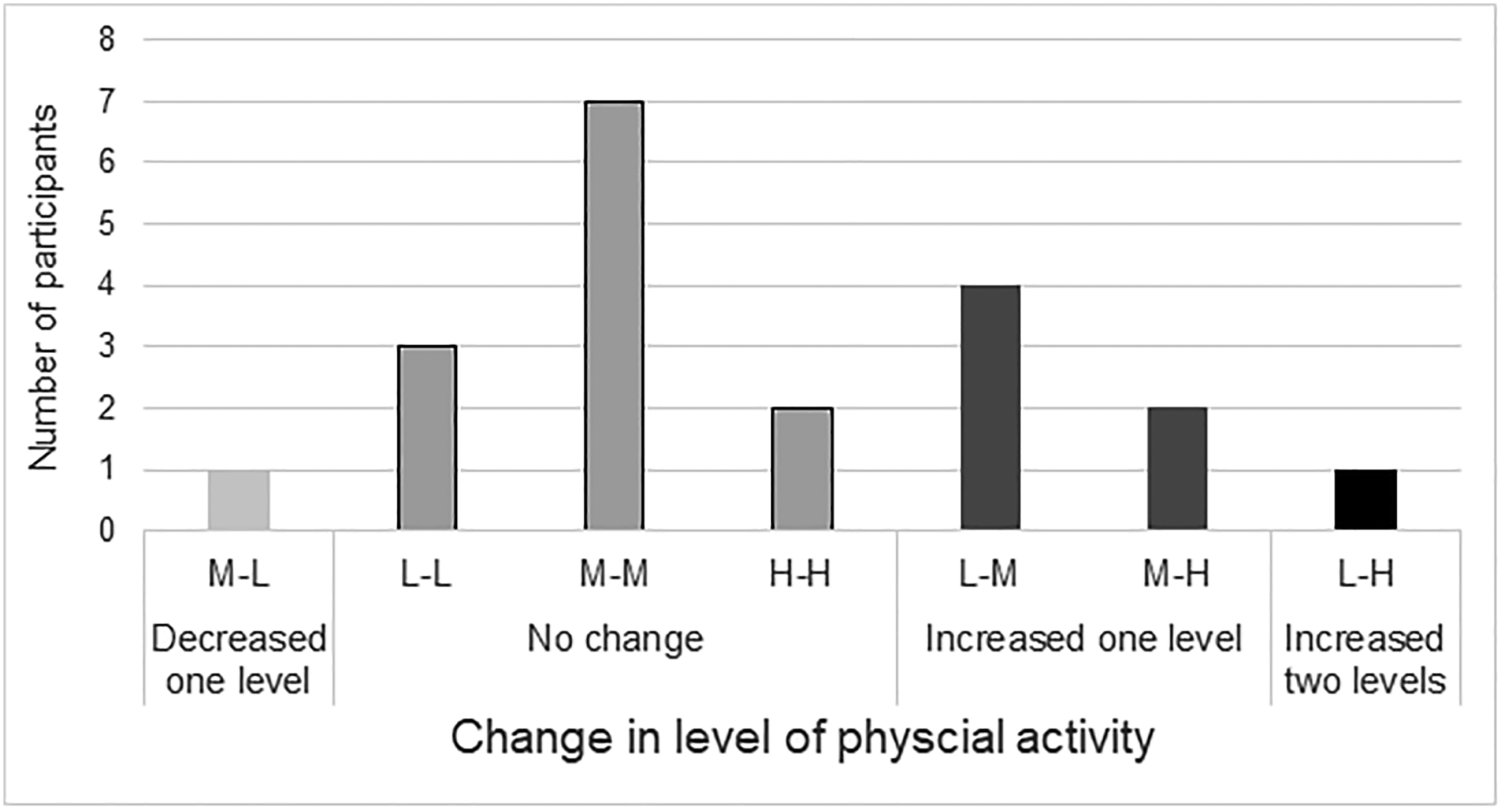
Level of physical activity from IPAQ. Number of participants who have decreased (light grey), had no change (grey), increased one level (dark grey) or increased two levels (black) of physical activity. Low activity level (L); Moderate activity level (M); High activity level (H). For example, the first bar M–L means that one participant has gone from Moderate (pre) to Low activity level (post).

Before the intervention period, 38.1% of the participants reported low PA levels, 52.4% reported moderate, and 9.5% reported a high PA level. After the intervention period, 20% of the participants reported that they had a low level of activity, 55% reported a moderate level, and 25% reported that they were highly active. Figure 1 presents the absolute changes in levels of PA. This was a significant increase in PA levels when comparing the pre‐intervention and post‐intervention period, *P* = 0.03.

Concerning quality of life, as evaluated with the MANSA, the mean score of the satisfaction items was 5.9 ± 0.92 pre‐training and 5.8 ± 0.9 post‐training, indicating non‐significant changes, *P* = 0.26, *R* = 0.06. One item showed a significant difference post‐training compared to pre‐training. This item was leisure activities, where the score was lower post‐training compared with pre‐training, *P* = 0,046.

The mean value of enjoyment of the training, evaluated with PACES, was 3.9 ± 1.1, with 5 being the highest value.

## Discussion

This study, targeting a vulnerable group, evaluated a new tool aimed at promoting the PA of people with ID by offering web‐based training to the participants. To our knowledge, no previous study has evaluated web‐based training for people with ID. It is therefore interesting to observe the attendance rate of the training of 83% and additionally the highly rated enjoyment (3.9 on a 5‐point scale). The enjoyment of the training is essential for maintaining training, and a previous study has shown that greater enjoyment of PA is associated with adherence to the training programme, a motivational factor that may have affected the observed results (Williams *et al*. 2006). The results of the current study are similar to previous studies of PA interventions that use traditional training and not web‐based training. A systematic review explored feasibility of training programmes in RCTs for people with ID, and it showed that the attendance rate ranged from 71% to 92.6% in four of those studies (Bouzas *et al*. 2019).

According to the WHO, the recommendation for PA is to be active at a moderate level of intensity for at least 150 min per week (Bull *et al*. 2020). Therefore, the results showing the increased PA‐level post‐training compared to pre‐training are promising with the results primarily showing fewer participants in low activity level and more with both a moderate and high level of PA. However, this was evaluated subjectively with a questionnaire and would have been objectively reliable if it had been evaluated objectively, for instance, with an accelerometer. However, the IPAQ‐SF has been shown to be reliable in obtaining similar results to objective ways of measuring PA (McKeon *et al*. 2013; Cleland *et al*. 2018). Furthermore, using pictures of PA in the questionnaire makes it easier for people with ID to take part in a questionnaire process along with assistance from caregivers (Reid *et al*. 2009).

When looking at the health parameters of BC and WC, which are an easy way to perform a risk assessment for metabolic syndrome and good predictors of CVDs, we observe positive changes. A decrease in fat mass indicates a negative energy balance during the intervention, that is, larger energy expenditure than energy intake. A decrease in fat mass will generally decrease the risk of type 2 diabetes and CVDs, such as heart attack and stroke (Emanuela *et al*. 2012). The risk for diseases increases with a waist circumference of higher than 94 cm for men and 80 cm for women. If the waist circumference is above 102 cm for men and over 88 cm for women, the risk becomes critical and a decrease in waist circumference is indicated (Ross *et al*. 2020). In this study, a decrease in waist circumference was observed; however, it did not reach the recommended values to minimise the risk for diseases, such as CVDs and type 2 diabetes. The women decreased their WC by a mean of 3.3 ± 4 cm, *P* = 0.03, from 98.6 to 95.3 cm, which is still above the health recommended values. However, this is a shift towards better health given that other factors affect WC, for example, diet (Wallén *et al*. 2013). A healthy diet is also of highly important for improving body composition; however, the present study focused on the level of PA alone and did not include control over the diet. A significant decrease in fat mass was also observed, although it was only significant for men, with a mean decrease of 2.4 ± 1.6 kg, *P* < 0.001. However, the results observed for the decrease in fat mass are still not within the recommended level of fat mass either. We can conclude that this is a short training period of 12 weeks and more extended studies and follow‐ups are needed to evaluate long‐term effects. Nevertheless, the current study's observed results are similar to or better than other studies have observed, for example, increased lean body mass and decreased BMI compared to a previous study with a similar population (Lee & Lee 2021). Given that group training with an instructor on‐site is often more motivational for performing training, and that this study evaluated online training that was not able to give the participants feedback, good improvements were observed.

With respect to QoL, no significant improvements were observed. However, a decrease in the scores for the item *leisure activities* was reported. Since this study was conducted during the COVID‐19 pandemic, pandemic‐related restrictions might have affected the extent to which participants had the opportunity to engage in leisure activities, as the post‐intervention time had a higher level of restrictions than the pre‐intervention time. According to previous research, the results observed from another study are inconsistent, and a variety of factors to measure QoL have been used (Bondar *et al*. 2020). The same systematic review mention that a number of barriers (transportation, assistance and social aspects) seem to affect the results of PA interventions on QoL. With the present study, transport was not a limitation, although both assistance in technical competence and the social aspects may still present a barrier. The observed changes may be explained by the fact that the participants enjoyed the type of training, which a previous study mention playing a role in motivation to participate in PA (Williams *et al*. 2006).

### Strength and limitations

The COVID‐19 pandemic might have had a positive effect on the study as many other possibilities were closed, such as doing other activities and the possibility to visit friends and family. This might have positively influenced participation in the study and contributed to the high attendance rate. The study did not control for food/drink intake, which may have affected the reliability of the BC (Brantlov *et al*. 2017). The reason was the assessed difficulty to control food and drink intake with high quality, for the participants (due to their disability) or their staff due to the participants' privacy. People with ID do in general have routines and schedules for the week and the day, which would imply similar food and drink intake at both pre‐measurement and post‐measurement. In the present study, PA was evaluated subjectively with a questionnaire, which is a weakness compared to objective measurements. However, this questionnaire has shown similar results as objective measurements of PA (McKeon *et al*. 2013; Cleland *et al*. 2018). A web‐based training programme overcomes some of the barriers to PA, such as transport issues, the lack of adapted options and financial limitations. However, barriers such as a lack of support from caregivers or parents and a lack of clear policies for involving regular PA are still important to overcome, despite the web‐based training.

## Conclusion

This study showed that a web‐based training programme, carried out three times a week for 12 weeks, for people with ID was effective and feasible for increasing PA levels and improving health markers for people with ID. No changes in QoL were observed. This is a new, innovative way of enhancing PA and overcomes some of the barriers to PA such as transportation and an unfamiliar environment. As such, the intervention seems promising for overcoming health‐equity concerns related to low levels of PA in people with ID. For middle‐income to high‐income countries where Internet connectivity is well established, web‐based training is a cheap and easy way for caregivers to involve PA in the daily schedule due to its flexibility and location independency.

## Source of funding

The study was funded by the Mid Sweden University agreement with the Municipality of Östersund.

## Conflict of interest

None.

## Ethics statement

The study was pre‐approved by the Swedish Ethical Review Authority (Dnr 2019‐06495 and Dnr 2020‐02607). No individual materials are presented in the paper. Research participants signed an informed consent before attending the study including information that data would be presented anonymous and using mean values. No materials from other sources are included.

## Supporting information


**Table S1:** Web‐based exercise intervention described in a Consensus on Exercise Template (CERT).

**Video S1:** A short example of the training programme.

## Data Availability

Individual data are not published online due to privacy for the participants.
