# Supplementary material for: Web‐based training intervention to increase physical activity level and improve health for adults with intellectual disability
Source: J Intellect Disabil Res. 2022 Oct 10;66(12):967–77. doi: 10.1111/jir.12984 (PMC9828805; doi:10.1111/jir.12984)
Supplement: Supplementary file 1 — Table S1: Web‐based exercise intervention described in a Consensus on Exercise Template (CERT). [file JIR-66-967-s002.docx]

| Section/Topic | Item # | Checklist Item | Location | |
| --- | --- | --- | --- | --- |
|  | | | Primary paper | Other |
| WHAT: materials | 1 | Detailed description of the type of exercise equipment  The equipment needed for the exercises was a computer/reading tablet or mobile phone to be able to watch the videos. No other exercise equipment was needed. A yoga mat/towel could be used if wanted to, for the exercises performed on the floor. | Page 7 (Intervention) | Supplementary video |
| WHO: provider | 2 | Detailed description of the qualifications, expertise and/or training undertaken by the exercise instructor  All training sessions were conducted digitally. The same instructor, who is a certified personal trainer with 20 years of experience, led all training sessions. Instructions from a special education teacher and a physiotherapist were given to the instructor in order to modify the training program for the target group. | Page 7 (Intervention) |  |
| HOW: delivery | 3 | Describe whether exercises are performed individually or in a group  The training was delivered by distance on a web-based platform. The participants could choose if they wanted to perform the exercises in a group or individually. They performed the training in their home and/or in a community facility where they live. The training sessions were pre-recorded. | Page 7 (intervention) |  |
|  | 4 | Describe whether exercises are supervised or unsupervised; how they are delivered  The exercise programme was unsupervised. However, the caregivers working with  207 the participants were invited to participate if they wanted to and assisted the participants with progression levels and intensity. | Page 8 (intervention) |  |
|  | 5 | Detailed description of how adherence to exercise is measured and reported  Participants, assisted by caregivers, filled in a protocol after they had participated in each completed training session. Attendance to the training sessions was calculated by dividing the number of training sessions attended by the number of training sessions scheduled. | Page 8 (intervention) |  |
|  | 6 | Detailed description of motivation strategies  Twice during the intervention period researchers held digital motivational talks with participants in groups, after four and eight weeks. The purpose of the talks was to identify any difficulties at an early stage and overcome the barriers together. | Page 8 (intervention) |  |
|  | 7 | Detailed description of the decision rule(s) for determining exercise progression  The goal was to reach a moderate intensity level. The intensity was applied using the OMNI-  Walk/Run Scale of Perceived Exertion, a rating of perceived exertion with 1 as the lowest and 10 the highest intensity. The participants could see pictures that described the different intensity levels and tried to reach 6-7 on the scale. Different progression levels were applied to be able to meet the participants’ requirements, e.g. they could choose from jumping/walking to be able to meet the moderate intensity level. | Page 8 (intervention) |  |
|  | 8 | Detailed description of each exercise to enable replication  Online supplementary appendices provided with video of the exercises. |  | Supplementary video |
|  | 9 | Detailed description of any home programme component | N/A |  |
|  | 10 | Describe whether there are any non-exercise components | N/A |  |
|  | 11 | Describe the type and number of adverse events that occur during exercise  No adverse events were reported during the exercises. However, four participants dropped out during the intervention period: one due to injury; the participant sustained a rib injury whilst  biking, one due to a lack of internet connection and two due to lack of motivation. | Page 11 (Results) |  |
| WHERE: location | 12 | Describe the setting in which the exercises are performed  All training sessions were pre-recorded and delivered through a commercially available platform MyMOWO (www.mymowo.com, Virtual Gym Sweden AG, Sweden). Participants performed the training sessions in their home or in a community facility where they live. | Page 7-8 (intervention) |  |
| WHEN, HOW MUCH: dosage | 13 | Detailed description of the exercise intervention  The training was divided into three classes, three times a week 50 min per session (2x25 min). A combination of strength and endurance exercise were included in the training programme along with balance and flexibility. |  | Supplementary video |
| TAILORING: what, how | 14a | Describe whether the exercises are generic (one size fits all) or tailored  The programme was modified for people with ID in cooperation with a special education teacher, a physiotherapist and the training instructors working with the commercial platform. | Page 7 (intervention) |  |
|  | 14b | Detailed description of how exercises are tailored to the individual  The adjustments made were 1) fewer instructions and talking from the instructor, 2) exercises that required easier coordination, 3) longer periods performing the same exercise before changing, 4) breaks with instructions to drink water and also the instructor drinking water, and 5) a timer showing the remaining time of the training session. | Page 7 (intervention) |  |
|  | 15 | Describe the decision rule for determining the starting level  Prior to the intervention the training programme were tested by persons with ID from a high school, to determine the appropriate starting level. | Page 7 (intervention) |  |
| HOW WELL: planned, actual | 16a | Describe how adherence or fidelity is assessed/measured  Participants, assisted by caregivers, filled in a protocol after they had participated in each completed training session, that measured adherence, defined as the number of sessions attended divided the number of sessions planned. | Page 8 (intervention) |  |
|  | 16b | Describe the extent to which the intervention was delivered as planned  Of the 28 participants, a total of 22 participants completed the study and performed web-based training for 12 weeks. These 22 participants performed a mean of 30 of the 36 planned training sessions, i.e. the mean adherence rate to the training sessions was 83%. | Page 11 (Results) |  |
